# Lactate dehydrogenase, an independent risk factor of severe COVID-19 patients: a retrospective and observational study

**DOI:** 10.18632/aging.103372

**Published:** 2020-06-24

**Authors:** Yi Han, Haidong Zhang, Sucheng Mu, Wei Wei, Chaoyuan Jin, Chaoyang Tong, Zhenju Song, Yunfei Zha, Yuan Xue, Guorong Gu

**Affiliations:** 1 Emergency Department, Zhongshan Hospital, Fudan University, Shanghai 20032, China; 2 Department of Radiology, Renmin Hospital of Wuhan University, Wuhan 430060, Hubei, China

**Keywords:** coronavirus disease 2019, COVID-19, lactate dehydrogenase (LDH), lung injury, inflammatory response

## Abstract

Background: The World Health Organization has declared coronavirus disease 2019 (COVID-19) a public health emergency of global concern. Updated analysis of cases might help identify the risk factors of illness severity.

Results: The median age was 63 years, and 44.9% were severe cases. Severe patients had higher APACHE II (8.5 vs. 4.0) and SOFA (2 vs. 1) scores on admission. Among all univariable parameters, lymphocytes, CRP, and LDH were significantly independent risk factors of COVID-19 severity. LDH was positively related both with APACHE II and SOFA scores, as well as P/F ratio and CT scores. LDH (AUC = 0.878) also had a maximum specificity (96.9%), with the cutoff value of 344.5. In addition, LDH was positively correlated with CRP, AST, BNP and cTnI, while negatively correlated with lymphocytes and its subsets.

Conclusions: This study showed that LDH could be identified as a powerful predictive factor for early recognition of lung injury and severe COVID-19 cases.

Methods: We extracted data regarding 107 patients with confirmed COVID-19 from Renmin Hospital of Wuhan University. The degree of severity of COVID-19 patients (severe vs. non-severe) was defined at the time of admission according to American Thoracic Society guidelines for community acquired pneumonia.

## INTRODUCTION

First reported in Wuhan, Hubei province, China, on December 2019, outbreak of a viral pneumonia has attracted extensive attention of international community [1]. The pathogen, a novel β-coronavirus, has currently been named severe acute respiratory syndrome coronavirus 2 (SARS-CoV-2) by the International Committee on Taxonomy of Viruses [2]. The World Health Organization (WHO) has recently declared coronavirus disease 2019 (COVID-19) a public health emergency of global concern [3]. As of March 1, 2020, more than 500,000 confirmed cases have been documented all over the world, with more than 50,000 severe cases, and a mortality rate of 4-15% [2, 4].

Among recent studies, the presence of any coexisting illness was more common among patients with severe disease [2]. Most of the patients had elevated levels of C-reactive protein, and lymphocytopenia was common, especially in severe cases [1, 2, 5], which was thought to be a result of reduction of CD3^+^, CD4^+^ and CD8^+^ T cells [6]. Meanwhile, prolonged prothrombin time (PT) and elevated lactate dehydrogenase (LDH) was found in more than 40% cases during the whole disease period [5, 7], with elevated ALT and AST less common in COVID-19 patients [2].

Given the rapid spread of COVID-19, we considered that an updated analysis of risk factors may help early recognition of the severity of the disease. In this study, we analyzed the clinical and laboratory parameters of severe and non-severe COVID-19 patients in order to evaluate disease severity.

## RESULTS

### Demographics and characteristics of COVID-19 patients

Diagnosis of COVID-19 was made according to World Health Organization interim guidance [8]. A total of 107 diagnosed cases were enrolled in this study, with 48 severe and 59 non-severe cases (Table 1). 60 cases (56.1%) were male, of which 64.4% were severe (*P* = 0.11). The median age was 63 years (IQR, 49-71 years), and severe patients were significantly older than the non-severe ones (67 vs. 61, *P* = 0.005). A total of 49 (45.8%) patients had underlying conditions, including hypertension (28 [26.2%]), diabetes (13 [12.1%]), coronary heart disease (5 [4.7%]), and autoimmune disease (3 [6.3%]). Although there was no significant difference in the underlying conditions between the two groups, there were more patients with coronary heart disease (3 [6.3%]) and autoimmune disease (3 [6.3%]) in the severe group than the non-severe group. All patients enrolled had fever, with maximum temperatures of approximately 39ºC of which 30 (28%) had fever with dyspnea. Severe patients had higher Acute Physiology and Chronic Health Evaluation (APACHE) II (8.5 vs. 4.0, *P* < 0.001) and Sequential Organ Failure Assessment (SOFA) (2 vs. 1, *P* < 0.001) scores on admission, as well as the higher Pneumonia Severity Index (PSI) (83.35 vs. 55.76, *P* < 0.001), CURB (Confusion/Urea/Respiratory rate/Blood pressure)-65 (1 vs. 0, *P* < 0.001) and computed tomography (CT) semiquantitative rating scores (4 vs. 1, *P* < 0.001).

**Table 1:** Demographic and characteristics of COVID-19 patients.

	**All patients (N = 107)**	**Nonsevere patients (N = 59)**	**Severe patients (N = 48)**	***P* value**
**Age(median, IQR)years**	63, (49-71)	61, (43-69)	67, (56-76)	0.005
**Gender**	-	-	-	0.110
**Male**	60, 56.1%	29, 49.2%	31, 64.4%	
**Female**	47, 43.9%	30, 50.8%	17, 35.4%	
**History**	-	-	-	
**Hypertension**	28, 26.2%	14, 23.7%	14, 29.2%	0.524
**DM**	13, 12.1%	9, 15.3%	4, 8.3%	0.276
**CHD**	5, 4.7%	2, 3.4%	3, 6.3%	0.655
**AD**	3, 2.8%	0, 0%	3, 6.3%	0.087
**Symptoms**	-	-	-	
**Fever(Highest) (median, IQR)°C**	38.3, (38-38.7)	38, (38-38.5)	38.4, (37.9-39)	0.478
**Dyspnea**	30, 28%	12, 20.3%	18, 37.5%	0.049
**APACHE II**	6.0, (3-8)	4.0, (2-6)	8.5, (6-11)	<0.001
**SOFA**	2, (1-2)	1, (0-1)	2, (2-3)	<0.001
**PSI**	68.1±30.7	55.76±24.53	83.35±30.95	<0.001
**CURB65**	1, (0-1)	0, (0-1)	1, (1-2)	<0.001
**CT score**	2,(1-4)	1, (1-2)	4, (3-5)	<0.001

(CHD: Coronary Heart Disease; AD : Autoimmune Disease)

### Laboratory indices of COVID-19 patients

Compared to the non-severe patients, neutrophil levels (*P* < 0.001), alanine transaminase (ALT) (*P* = 0.001), aspartate transaminase (AST) (*P* < 0.001), LDH (*P* < 0.001), Urea (*P* = 0.006), C-reactive protein (CRP) (*P* < 0.001), troponin I (cTnI) (P < 0.001), creatine kinase-MB (CKMB) (*P* < 0.001), B-type natriuretic peptide (BNP) (*P* < 0.001), prothrombin time (PT) (*P* < 0.001), activated partial thromboplastin time (APTT) (*P* = 0.022), and D-dimer (*P* < 0.001) in severe patients were significantly higher at admission. Conversely, lymphocyte (*P* < 0.001), monocyte (*P* < 0.001), CD3^+^ (*P* < 0.001), CD4^+^ (*P* < 0.001), CD8^+^ (*P* < 0.001), CD19^+^ (*P* = 0.015) and CD16^+^56^+^ (*P* = 0.010) T cells in severe patients were significantly lower, as well as PaO_2_/FiO_2_ (P/F) ratio (*P* < 0.001) (Table 2). No significant differences in the serum levels of immunoglobulins (IgA, IgE, IgG and IgM) or complement C3 and C4 were observed between the two groups (Table 2).

**Table 2:** Laboratory Indices of COVID-19 patients.

	**All patients (N = 107)**	**Nonsevere patients (N = 59)**	**Severe patients (N = 48)**	***P* value**
**White blood cell (×10^9/L)**	5.81, (4.23-8.11)	5.38, (4.23-7.26)	7.11, (4.22-10.4)	0.032
**Neutrophil (×10^9/L)**	3.88, (2.52-6.08)	3.41, (2.14-4.64)	5.67, (3.22-9.57)	<0.001
**Lymphocyte (×10^9/L)**	1.0, (0.68-1.5)	1.3, (1.01-1.71)	0.72, (0.58-0.92)	<0.001
**Monocyte (×10^9/L)**	0.46±0.21	0.54±0.21	0.35±0.17	<0.001
**CD3(/uL)**	550, (343-846.5)	819.5, (572-1083.5)	365, (289-527.5)	<0.001
**CD4(/uL)**	336, (226.5-539.5)	509, (350-697.75)	240, (172-317)	<0.001
**CD8(/uL)**	191, (104.5-311.5)	259.5, (191.25-366.5)	118, (64-187)	<0.001
**CD19(/uL)**	123, (81-197)	175.5, (101.25-226.25)	102, (76-167)	0.015
**CD16+56(/uL)**	115, (70.5-186.5)	135.5, (97.75-227.5)	95, (61.5-161.5)	0.010
**ALT(U/L)**	24, (17-39)	19.5, (16-29.5)	29, (21-51)	0.001
**AST(U/L)**	27, (19-39)	21, (17.25-27)	39, (29-56)	<0.001
**ALB(g/L)**	37.91±5.95	40.18±5.84	34.95±4.68	<0.001
**TB(umol/L)**	12, (8.5-15.7)	10.8, (8.2-14.4)	13.6, (9.4-17.3)	0.037
**LDH(U/L)**	273, (195-414)	206, (174-272)	426, (298.25-516.25)	<0.001
**Urea (mmol/L)**	4.76, (3.98-6.51)	4.39, (3.82-5.84)	6.3, (4.2-8.4)	0.006
**Crea (umol/L)**	64, (53-74)	64.5, (53-73.5)	63, (54-74)	0.846
**BG (mmol/L)**	5.46, (4.78-6.93)	5.12, (4.61-6.0)	6.12, (5.26-7.54)	0.003
**CRP (mg/L)**	35.8, (5.0-85.1)	6.35, (5.0-38.18)	93.4, (37.73-158.38)	<0.001
**cTnI (ng/mL)**	0.006, (0.006-0.011)	0.006, (0-0.006)	0.01, (0.006-0.073)	<0.001
**CKMB (ng/mL)**	1.16, (0.64-1.85)	0.96, (0.62-1.33)	1.48, (0.91-2.96)	<0.001
**BNP (pg/mL)**	147, (50.43-437.7)	84.33, (22.64-170.68)	289, (135-911.2)	<0.001
**PT (s)**	12, (11.28-13)	11.8, (11.2-12.3)	12.4, (11.85-13.45)	<0.001
**APTT (s)**	28.05, (26.1-30.6)	26.9, (25.85-29.65)	28.8, (26.3-32.1)	0.022
**D-Dimer (mg/L)**	0.76, (0.36-2.14)	0.45, (0.26-1.04)	2.06, (0.76-8.37)	<0.001
**Fib (g/L)**	4.54±1.57	4.05±1.53	5.21±1.37	<0.001
**IgA (g/L)**	2.28, (1.76-3.03)	2.07, (1.65-2.63)	2.65, (1.83-3.97)	0.053
**IgE (IU/mL)**	89.4, (37.8-157.5)	73.8, (31.7-128)	97.65, (38.42-272)	0.374
**IgM (g/L)**	0.96±0.40	1.0±0.44	0.91±0.36	0.279
**IgG (g/L)**	11.7, (9.97-13.2)	11.65, (9.95-12.7)	11.9, (10.01-15.2)	0.252
**C3 (g/L)**	1.04±0.25	1.03±0.23	1.07±0.28	0.467
**C4 (g/L)**	0.27±0.12	0.26±0.15	0.28±0.08	0.546
**PaO2/FiO2 (mmHg)**	250.26±142.87	413.22±141.31	214.01±116.89	<0.001

### Independent risk factors of severe COVID-19 patients

To assess the risk factors of the demographics, characteristics, and laboratory indicators on the severity of COVID-19 patients, logistic regression analysis was performed on the parameters of significant difference using *t* test. In univariable analysis, odds ratio of serum CKMB concentration and PT level were the highest in severe patients. Male patients infected with SARS-CoV-2 showed as an independent risk factor for being in a more severe condition as 1.89 (0.86-4.12). Apart from the risk factors above, patient age, white blood cell count, neutrophil count, serum AST, ALT, LDH, Urea, CRP, and D-dimer level were all associated with the severity of COVID-19 patients. Meanwhile, we found that the lymphocytes, monocytes, CD3^+^, CD4^+^, CD8^+^, CD19^+^ T cells and P/F ratio were protective factors (OR < 1) for COVID-19 patients. Based on the condition we mentioned, age, gender, dyspnea, and laboratory indicators of lymphocytes, CRP, cTnI and LDH were chosen for a multivariable logistic regression model. As a result, serum lymphocytes (OR:0.2, 95% CI:0.04-0.96, *P* < 0.05), CRP (OR:1.026, 95% CI:1.006-1.046, *P* < 0.05), and LDH (OR:1.009, 95% CI:1.002-1.016, *P* < 0.05) were found to be independent risk factors for the severity of COVID-19 patients (Table 3).

**Table 3:** Univariable OR and multivariable OR of severe COVID-19 patients

	**Univariable OR (95% CI)**	**P value**	**Multivariable OR (95% CI)**	**P value**
**Demographics and clinical characteristics**				
**Age(years)**	1.04 (1.01-1.07)	0.005	0.99 (0.94-1.05)	0.786
**Gender(Male)**	1.89 (0.86-4.12)	0.111	0.40 (0.09-1.87)	0.244
**Dyspnea**	2.35 (0.99-5.57)	0.052	2.51 (0.59-10.62)	0.212
**Labaray paremeters**				
**White blood cell (×10^9/L)**	1.20 (1.04-1.38)	0.011		
**Neutrophil (×10^9/L)**	1.34 (1.14-1.57)	<0.001		
**Lymphocyte (×10^9/L)**	0.019 (0.009-0.11)	<0.001	0.20 (0.04-0.96)	0.044
**Monocyte (×10^9/L)**	0.005 (0.00-0.067)	<0.001		
**CD3(/uL)**	0.995 (0.992-0.997)	<0.001		
**CD4(/uL)**	0.992 (0.988-0.995)	<0.001		
**CD8(/uL)**	0.989 (0.984-0.994)	<0.001		
**CD19(/uL)**	0.994 (0.989-0.999)	0.017		
**CD16+56(/uL)**	0.996 (0.991-1.00)	0.076		
**ALT(U/L)**	1.03 (1.01-1.05)	0.005		
**AST(U/L)**	1.10 (1.05-1.14)	<0.001		
**LDH(U/L)**	1.01 (1.01-1.02)	<0.001	1.009 (1.002-1.016)	0.016
**Urea(mmol/L)**	1.08 (0.97-1.20)	0.167		
**BG (mmol/L)**	1.18 (0.99-1.41)	0.060		
**CRP (mg/L)**	1.03 (1.02-1.05)	<0.001	1.026 (1.006-1.046)	0.012
**cTnI (ng/mL)**	7832.09 (0.29-2.1x10^9)	0.085	1.803 (0-132491.35)	0.918
**CKMB (ng/mL)**	2.18 (1.28-3.73)	0.004		
**BNP (pg/mL)**	1.00 (1.00-1.00)	0.638		
**PT (s)**	2.50 (1.49-4.19)	0.001		
**APTT (s)**	1.17 (1.02-1.33)	0.022		
**D-Dimer (mg/L)**	1.61 (1.14-2.27)	0.007		
**Fib (g/L)**	1.70 (1.24-2.31)	0.001		
**PaO2/FiO2 (mmHg)**	0.988 (0.980-0.997)	0.005		

### The predictive factors correlated with severity of COVID-19

**Figure 1:**
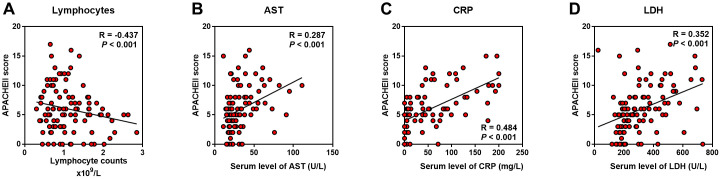
**Predictive factors correlated with severity of COVID-19 patients.** Correlation analysis was performed between candidate indicators with APACHE II score. (**A**) Lymphocyte counts was negatively correlated with APACHE II; (**B**–**D**) AST, CRP and LDH were positively correlated with APACHE II.

We used clinical severity scores (APACHE II and SOFA) to assess the disease severity in COVID-19 patients. The average APACHE II score was 8.5 in severe cases versus 4.0 in non-severe cases, with SOFA 2.0 versus 1.0 (Table 1, *P* < 0.001). We used speculated factors such as lymphocytes, AST, CRP and LDH performing Pearson and Kendall’s tau_b correlation analysis with APACHE II and SOFA score. The results showed that lymphocytes had a negative correlation with APACHE II (R = -0.437, *P* < 0.001) and SOFA (R = -0.486, *P* < 0.001), while other indicators CRP (R = 0.484 *P* < 0.001, R = 0.580 *P* < 0.001), LDH (R = 0.352 *P* < 0.001, R = 0.560 *P* < 0.001) and AST (R = 0.287 *P* < 0.001, R = 0.425 *P* < 0.001) were positively associated with both APACHE II and SOFA scores (Figure 1, Table 4).

**Table 4:** Correlation with SOFA and CT semiquantitative rating score.

**Predictive factors**	**SOFA score**		**CT score**	
	**Correlation coefficient**	***P* value**	**Correlation coefficient**	***P* value**
**Lymphocyte (×10^9/L)**	-0.486	<0.001	-0.411	<0.001
**AST (U/L)**	0.425	<0.001	0.517	<0.001
**LDH (U/L)**	0.560	<0.001	0.556	<0.001
**CRP (mg/L)**	0.580	<0.001	0.507	<0.001

### The predictive factors correlated with the severity of lung damage

**Figure 2:**
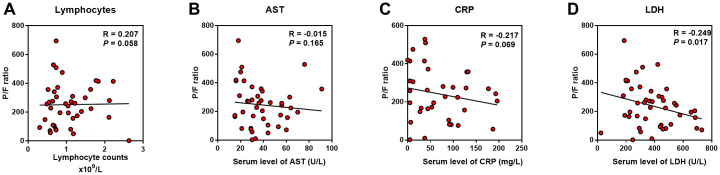
**Predictive factors correlated with lung injury of COVID-19 patients.** Correlation analysis was performed between the indicators with P/F ratio. (**A**–**C**) Lymphocyte counts, AST and CRP were not correlated with P/F ratio; (**D**), LDH was negatively correlated with P/F ratio.

We used P/F ratio to assess the severity of pneumonia induced lung injury in COVID-19 patients. The average P/F ratio was 214 mmHg in severe cases versus 413 mmHg in non-severe cases (Table 2, *P* < 0.001). We also evaluated the extent of inflammation on chest CT using a semiquantitative rating system (Supplementary Table 1); the median score was 4.0 in severe cases while only 1.0 in non-severe cases (Table 1, *P* < 0.001). Indicators above were further performed Pearson and Kendall’s tau_b correlation analysis with P/F ratio and CT rating score to determine the potential biomarkers for the lung injury. As a result, the serum LDH level showed the highest R value, positively with CT score (R = 0.556, *P* < 0.001) and negatively with P/F ratio (R = -0.249, *P* = 0.017) in all the indicators (Figure 2D, Table 4). Nevertheless, although the serum CRP (R = 0.507, *P* < 0.001), AST (R = 0.519, *P* < 0.001) and lymphocytes (R = -0.411, *P* < 0.001) were significantly related to CT scores, they showed no correlation with P/F ratio (Figure 2A–2C).

### The predictive factors for identification of severe COVID-19 cases

**Figure 3:**
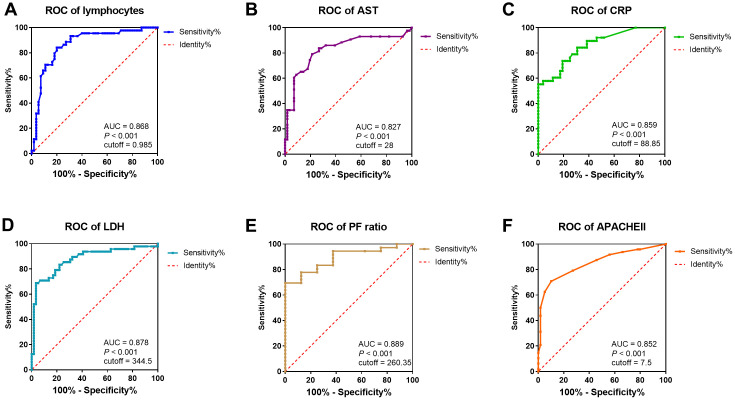
**ROC curve and cutoff value of predictive factors.** The factors for the prediction of COVID-19 patients getting severe condition. (**A–F**) ROC curve of lymphocytes, AST, CRP, LDH, P/F ratio, and APACHE II. AUC, area under curve.

To assess the diagnostic value of these selected parameters, receiver operating characteristic (ROC) curve and area under ROC curve (AUC) were calculated using R package “pROC”. As indicated in Figure 3, the area under curve (AUC = 0.878) implied a perfect accuracy of the serum LDH level more than 344.5 U/L in COVID-19 patients as a predictive factor for identification of severe condition, with the high specificity (96.9%) and sensitivity (68.8%) (Figure 3D). The serum AST level over 28 U/L and CRP over 88.85 mg/L showed relative moderate accuracy with AUC = 0.827 and AUC = 0.859 (Figure 3B, 3C). As a protective factor, the lymphocytes less than 0.985 x 10^9^ /L showed a good accuracy for identification of severe patients with AUC = 0.868, the maximum specificity (84.1%) and sensitivity (80.0%) (Figure 3A). Furthermore, AUC of P/F ratio was 0.889, CT score was 0.881, and APACHE II was 0.852. The other indicators were relatively poor accuracy factors in ROC curve analysis (Figure 3, Table 5).

**Table 5:** Cutoff value of predictive factors.

	**Sensitivity**	**Specificity**	**Cutoff value**	**AUC**	**P value**
**Lymphocytes (×10^9/L)**	0.80	0.841	0.985	0.868	<0.001
**CRP (mg/L)**	0.553	1.0	88.85	0.859	<0.001
**LDH (U/L)**	0.688	0.966	344.5	0.878	<0.001
**AST (U/L)**	0.791	0.786	28	0.827	<0.001
**P/F Ratio (mmHg)**	1.0	0.694	260.35	0.889	<0.001
**CT score**	0.818	0.857	2.5	0.881	<0.001
**APACHE II**	0.708	0.898	6.5	0.852	<0.001
**PSI**	0.604	0.814	76.5	0.757	<0.001

### Relationship between LDH and inflammation, cardiac and liver injury biomarkers

**Figure 4:**
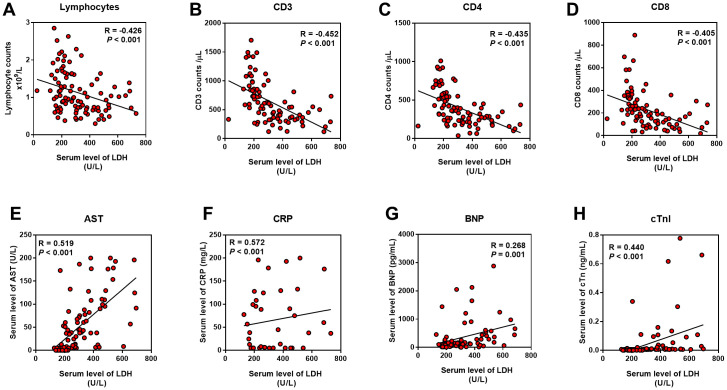
**Relationship between LDH and inflammation, cardiac and liver injury.** Pearson correlation analysis was performed between the indicators with the serum LDH level. (**A**–**D**) LDH was negatively correlated with lymphocyte and its subsets; (**E**–**H**) LDH was positively correlated with AST, CRP, BNP and cTnI.

As the serum LDH level showed the highest Correlation Coefficient in the correlation with APACHE II, SOFA, CT score, and P/F ratio, we evaluated the relationship between LDH and lymphocytes (including subsets), serum CRP, AST, BNP, and cTnI level. We found that LDH was positively correlated with CRP, AST, BNP, and cTnI, while negatively correlated with lymphocytes and its subsets, including CD3^+^, CD4^+^ and CD8^+^ T cells (P < 0.01) (Figure 4).

## DISCUSSION

In this study, we analyzed the clinical features in 107 patients with COVID-19 who were admitted to Renmin Hospital of Wuhan University between February 1 and March 1, 2020. Although the clinical characteristics of patients enrolled were somewhat akin to those reported in previous studies [1, 5, 7], there were no differences in gender and proportion with underlying diseases between severe and non-severe patients. Patients with advanced age were more likely to progress into severe pneumonia, which was not unexpected, in concert with recent studies [9]. We also found that the clinical characteristics of COVID-19 mimic those of SARS-CoV [1, 5, 10]. Fever and cough were the dominant symptoms, both groups had similar maximum temperature and number of patients who had dyspnea. APACHE II and SOFA scores were calculated based on admission data. PSI, CURB-65, and the CT semiquantitative rating score were used to assess the severity of lung damage. Significantly higher scores were found in severe cases.

Among the risk factors we investigated in this study, we surprisingly discovered that LDH had the most positive relationship between both P/F ratio and CT score. In addition, it was also most positively relevant to APACHE II and SOFA scores, which reflected a strong correlation between LDH with lung damage as well as disease severity. LDH is a major player in glucose metabolism which is present in tissues throughout the body and catalyzes pyruvate to lactate. It is released from cells upon damage of their cytoplasmic membrane [11]. Previous studies also had noted the importance of LDH as an indicator of lung diseases. In a study on Epstein-Barr virus (EBV), researchers found that EBV infected B cells had more LDH transcripts than the uninfected B cells [12]. In addition, the serum levels of LDH increased in pneumocystis pneumonia (PcP) patients, probably was due to lung injury [13, 14]. Among patients who were infected during the 2009 influenza A (H1N1) pandemic, 77.8% whose laboratory test showed LDH > 225U/L had lung involvement, without differences between adult and children [15], which indicated that LDH elevation was associated with various pathogens including viruses, and was relevant to lung injury. Furthermore, there was a case reported in 2017 that a patient with human Zika virus infection had markedly elevated LDH, which was associated with 70% mortality in further a Zika-infected animal study. They considered LDH as an indicator of multiorgan injury, not only affecting liver or cardiac function [16].

LDH is found in all human cells, especially in myocardial and liver cells. In our study, LDH elevation was positively associated with AST, cTnI and BNP, which verified it as an isozyme of heart and liver. However, it was somewhat surprising that cTnI and BNP were not associated with P/F ratio, while was relevant with disease severity (data not shown). Exactly similar with which, AST, that was associated with the APACHE II and SOFA score, was not related to P/F ratio, either. This rather intriguing finding might be explained by the fact that the myocardial and liver injury caused by SARS-CoV-2 might be due to the direct damage of the virus to targeted organs, not because of hypoxia induced by lung injury. Since the outbreak of COVID-19, structural analysis of the virus has suggested that SARS-CoV-2 might be able to bind to the angiotensin-converting enzyme 2 (ACE2) receptor in humans [17, 18]. The ACE2 receptor is abundantly present in the epithelia of lung and small intestine [19], which might provide possible routes of entry for SARS-CoV-2. This epithelial expression, together with its presence in vascular endothelium [19], also provides a step in understanding the pathogenesis of ARDS, cardiac injury, liver injury, and even MODS.

Furthermore, LDH was found to be positively associated with CRP and negatively with lymphocytes. An increase in CRP and decrease in lymphocytes were observed in severe cases during the 14-day observation period, which was consistent with findings of recent reports [1, 5, 6]. In our study, the development of lymphopenia in severe patients was mainly related to the significantly decreased absolute counts of T cells, especially CD3^+^, CD4^+^, and CD8^+^T cells, but not to B cells or NK cells. The decrease of T cells in severe cases reached its trough within three days, and then slightly increased from the first week while still maintaining low levels and not recovering to the level of non-severe patients over two weeks (Supplementary Figure 1).

LDH is not only a metabolic but also an immune surveillance prognostic biomarker, its elevation is a harbinger of poor outcomes in immunosuppressed patients [20]. LDH increases production of lactate, leads to enhancement of immune-suppressive cells, including macrophages and dendritic cells (DCs), and inhibition of cytolytic cells, such as natural killer (NK) cells and cytotoxic T-lymphocytes (CTLs) [11]. LDH is often induced upon T cell activation and proliferation [21, 22]. In a retrospective analysis of a CTLs antigen-4 antibody which could enhance T-cell activity and proliferation, the results showed that an increase in LDH level was indicative of a poor outcome [23], which confirms the inhibition effect of LDH on CTLs. Furthermore, CD4^+^ T cells produce less IFN-γ in the absence of LDH, demonstrating a critical role for LDH in promoting T cell responses [22].

It was also hypothesized that change in lactate modulated the inflammatory response in macro-phages [24]. Suppression of LDH has anti-inflammatory effects due to the downregulation of several inflammatory mediators including cytokines and NO [24]. Also, significant correlations were found between LDH and cytokines/chemokines, therefore suggesting that LDH may be a useful biomarker to assist the clinician in the decision to hospitalize a child with bronchiolitis [25]. In our study, lymphocytes, especially CD3^+^, CD4^+^, and CD8^+^ T cells were significantly decreased and relevant with LDH elevation. The decrease in T cell counts was strongly correlated with the severity of disease, which was in keeping with previous studies on SARS [26, 27]. On the other hand, elevation of LDH, the immune-related factor, could be considered as a predictive factor, that reflected a poor prognosis in severe COVID-19 patients.

Our study had some limitations. This study was conducted at a single-center with limited sample size. Furthermore, because many patients remained in hospital and outcomes were unknown at the time of writing, we only collected clinical data within two weeks for our analysis. COVID-19 has spread rapidly and has a wide spectrum of severity. A larger cohort study of patients with COVID-19 globally would help to further define the clinical characteristics and risk factors of the disease.

## CONCLUSION

In summary, this study showed that LDH could be identified as a powerful predictive factor for early recognition of lung injury and severe COVID-19 cases. And importantly, lymphocytes, especially CD3^+^, CD4^+^, and CD8^+^ T cells in the peripheral blood of COVID-19 patients, which was relevant with serum LDH, were also dynamically correlated with the severity of the disease.

## MATERIALS AND METHODS

### Data collection

107 confirmed COVID-19 patients at Renmin Hospital of Wuhan University between February 1 to March 1, 2020 were enrolled into this retrospective observational study. A confirmed case of COVID-19 was defined as a positive result on real-time reverse-transcriptase-polymerase-chain-reaction (RT-PCR) assay of nasal-pharyngeal swab specimens.

A trained team of physicians and medical students reviewed and collected demographic, epidemiological, clinical, physical examination findings, and laboratory data from electronic medical records. Laboratory assessments consisted of complete blood count, liver and renal function, markers of cardiac injury, measures of electrolytes, C-reactive protein and procalcitonin, and assessment of coagulation and lactate dehydrogenase, among other parameters. We defined the degree of severity of COVID-19 patients (severe vs. non-severe) at the time of admission, according to American Thoracic Society (ATS) guidelines for CAP [28]. If imaging scans were available, the radiologic assessments of chest computed tomography (CT) were reviewed and scored by an experienced senior radiologist who extracted the data. The APACHE II and SOFA score were calculated based on clinical and experimental data on admission, and CT score was calculated based on a semiquantitative rating system (Supplementary Table 1).

Patients were followed up for 14 days after admission. Patient information was confidentially protected by assigning a deidentified ID to each patient. The study was approved by the Ethics Committee of Renmin Hospital of Wuhan University.

### Statistical analysis

A sample size of at least 17 patients per group is needed to achieve 91% power to detect a difference of 0.3 between the area under the ROC curve (AUC) under the null hypothesis of 0.5 and an AUC under the alternative hypothesis of 0.8 using a two-sided test at a significance level of 0.05 (PASS 15, NCSS, LCC).

Because the patients enrolled in our study were not randomly assigned, all statistical findings should be interpreted as descriptive only. Quantized variables were presented as means ± standard deviation, and significance was tested by t-test. Nonparametric variables were expressed as medians and interquartile ranges or simple ranges as appropriate, and we used the Mann Whitney U or Kruskal Wallis tests to compare differences. Continuous and categorical variables were summarized as counts and percentages, and significance was detected by chi square or Fisher’s exact test. To explore the risk factors associated with severity of COVID-19, univariable and multivariate logistic regression models were used. Correlation analysis was performed by using Pearson and Kendall’s tau_b Correlation Coefficient. The sensitivity and specificity of the risk factors for the patient diagnosis were represented and analyzed by receiver operating characteristic curve (ROC curve). All the analyses and figures were performed with SPSS software (Version 26) and Graphpad Prism (Version 7.0). *P* < 0.05 was considered statistically significant in all analyses.

## Supplementary Material

Supplementary Figure 1

Supplementary Table 1
