# Supplementary material for: Lactate dehydrogenase, an independent risk factor of severe COVID-19 patients: a retrospective and observational study
Source: Aging (Albany NY). 2020 Jun 24;12(12):11245–58. doi: 10.18632/aging.103372 (PMC7343511; doi:10.18632/aging.103372)
Supplement: Supplementary Figure 1 [file aging-12-103372-s002..pdf]

SUPPLEMENTARY FIGURE

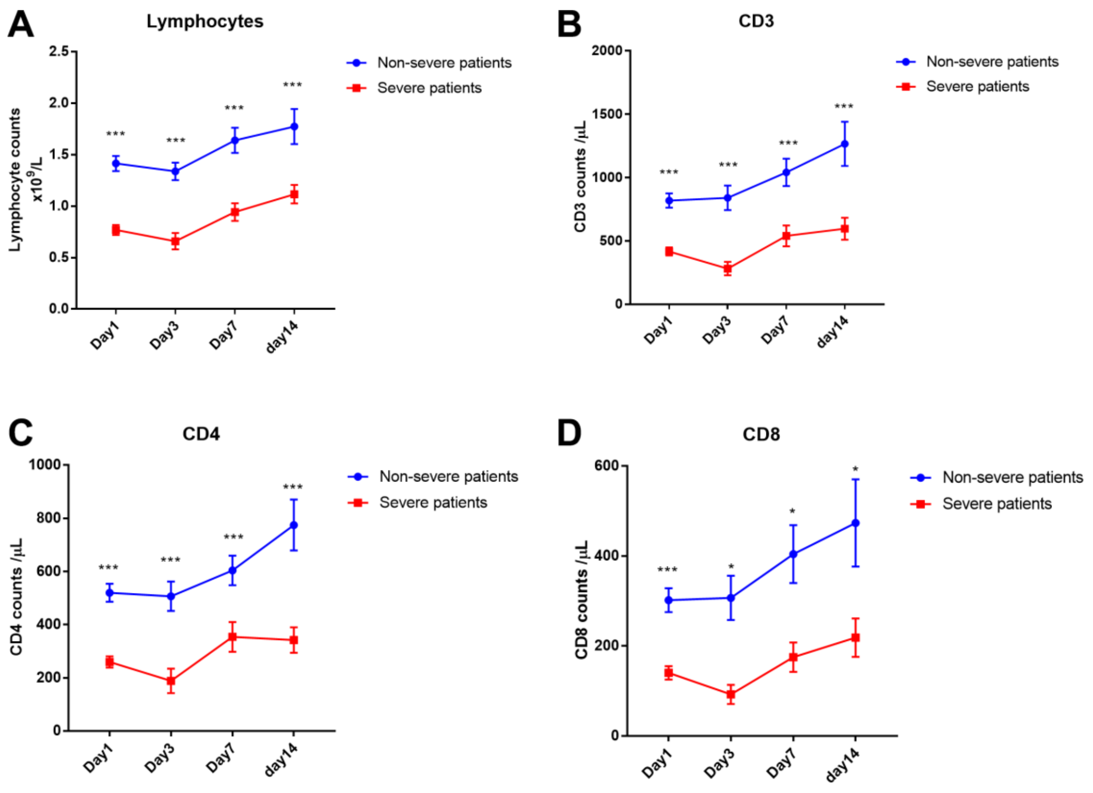

Supplementary Figure 1. Change of lymphocyte and its subsets during 14-day observation period. (In severe cases, the decrease of cells reached its trough within three days, and then slightly increased from the first week while still maintaining low levels and not recovering to the level of non-severe patients over two weeks. (**A**) change of lymphocyte; (**B**–**D**) change of lymphocyte subsets.).
