# Supplementary material for: Lactate dehydrogenase, an independent risk factor of severe COVID-19 patients: a retrospective and observational study
Source: Aging (Albany NY). 2020 Jun 24;12(12):11245–58. doi: 10.18632/aging.103372 (PMC7343511; doi:10.18632/aging.103372)
Supplement: Supplementary Table 1 [file aging-12-103372-s001..pdf]

SUPPLEMENTARY TABLE

Supplementary Table 1. Semiquatitative rating system based on CT image.

Methods	Based on Lobes		Based on Whole Lung
	6-part	5-part	
**Evaluation Range**	0, 1, 2, 3, 4	0, 1, 2, 3, 4, 5	Disease dimension/whole lung dimension (%)
**Evaluation Standard**	Based on the lobes involved		Based on whole lung
	0, % lesion<br>1, <25%<br>2, 26-49%<br>3, 50-75%<br>4, >75%	0, 0% lesion<br>1, <5%<br>2, <25%<br>3, 26-49%<br>4, 50-75%<br>5, >75%	Calculate the mean, maximum and minimum CT value of lesions
**Advantage**	Easy	Standard	More quantitative
